# Serum proteomic test in advanced non-squamous non-small cell lung cancer treated in first line with standard chemotherapy

**DOI:** 10.1038/bjc.2016.387

**Published:** 2016-11-29

**Authors:** F Grossi, E Rijavec, C Genova, G Barletta, F Biello, C Maggioni, G Burrafato, C Sini, M G Dal Bello, K Meyer, J Roder, H Roder, J Grigorieva

**Affiliations:** 1 Lung Cancer Unit, IRCCS AOU San Martino – IST Istituto Nazionale per la Ricerca sul Cancro, Largo R. Benzi 10, 16132 Genova, Italy; 2 Biodesix, 2970 Wilderness Place, Boulder, CO 80301, USA

**Keywords:** non-small cell lung cancer, VeriStrat, chemotherapy, biomarkers, prognosis, cisplatin, carboplatin, pemetrexed

## Abstract

**Background:**

VeriStrat is a blood-based proteomic test with predictive and prognostic significance in second-line treatments for non-small cell lung cancer (NSCLC). This trial was designed to investigate the role of VeriStrat in first-line treatment of advanced NSCLC with standard chemotherapy. Here we present the results for 76 non-squamous patients treated with a combination of carboplatin or cisplatin with pemetrexed.

**Methods:**

The test-assigned classifications of VeriStrat Good or VeriStrat Poor to samples collected at baseline. The primary end point was progression-free survival (PFS); secondary end points included overall survival (OS) and objective response. Exploratory analyses of end points separately in carboplatin/pemetrexed and cisplatin/pemetrexed subgroups were also conducted.

**Results:**

Patients classified as VeriStrat Good had longer PFS and OS than VeriStrat Poor: 6.5 *vs* 1.6 months and 10.8 *vs* 3.4 months, respectively; the corresponding hazard ratios (HRs) were 0.36 (*P*<0.0001) and 0.26 (*P*<0.0001); they were also more likely to achieve objective response. Prognostic significance of VeriStrat was confirmed in multivariate analysis. Significant differences in OS and PFS between Veristrat classifications were also found when treatment subgroups were analysed separately.

**Conclusions:**

The trial demonstrated clinical utility of VeriStrat as a prognostic test for standard first-line chemotherapy of non-squamous advanced NSCLC.

Non-small cell lung cancer (NSCLC) is one of the major causes of cancer-related death worldwide. The 5-year survival rate depends markedly on stage at diagnosis, from 49 to 16 to 2% for patients with local, regional, and distant-stage disease, respectively (Ries *et al*, 2005). There is no cure for patients with stage IV NSCLC, and the therapeutic goal for such patients is the prolongation of survival while alleviating symptoms and improving quality of life. The choice of first-line treatment depends on clinicopathological characteristics, such as Eastern Cooperative Oncology Group (ECOG) performance status (PS), age, histology, comorbidity, and molecular genetic features. In the case of the 10–15% of lung cancers harbouring epidermal growth factor receptor (EGFR) mutations (in the Caucasian population) and another 3–5% having anaplastic lymphoma kinase (ALK) rearrangements, targeted therapy with erlotinib, gefitinib, or afatinib in the former and with crizotinib in the latter case is recommended. In the absence of targetable mutations, platinum-based doublet chemotherapies remain the mainstay of treatment of newly diagnosed patients (Reck *et al*, 2014; Masters *et al*, 2015). Several doublet regimens with third-generation agents, such as paclitaxel, gemcitabine, docetaxel, vinorelbine, and pemetrexed have shown comparable efficacy (Delbaldo *et al*, 2007). However, the benefit from these therapies remains modest, and patients receiving pemetrexed and a platinum derivate as first-line treatment for non-squamous NSCLC currently achieve a median time to progression ∼4–6 months and median survival ∼9–15 months (Scagliotti *et al*, 2008; Rodrigues-Pereira *et al*, 2011; Moro-Sibilot *et al*, 2015).

Optimisation of chemotherapy treatment would be possible with the discovery and utilisation of reliable molecular biomarkers, and several candidates such as expression of the excision repair cross-complementation group 1 protein and ribonucleotide reductase subunit M1 as biomarkers for platinum chemotherapy (Azuma *et al*, 2007; Martin *et al*, 2008) and thymidylate synthase as a biomarker for pemetrexed (Nicolson *et al*, 2013; Sun *et al*, 2015), were suggested. However, insufficient tissue, problems with the method's reproducibility, paucity of validated biomarker trials, as well as often discordant protein expression between primary tumour and metastatic sites have resulted in a lack of validated tests for cytotoxic therapy in broad clinical practice. Non-invasive prognostic and predictive tests for standard chemotherapy regimens are highly desirable.

VeriStrat (Biodesix Inc., Boulder, CO, USA) is a commercially available blood-based proteomic mass spectrometry test developed for assessing clinical outcome following EGFR – tyrosine kinase inhibitor (EGFR TKI) therapy in patients with advanced NSCLC (Taguchi *et al*, 2007). VeriStrat is a true multivariate test measuring and analysing multiple components simultaneously, reflecting the complexity of the host–tumour interactions implicated in treatment outcomes. The details of the biological mechanism related to outcomes in the VeriStrat groups are yet unknown; however, some mass spectral features associated with the test strongly correlate with acute-phase reactants such as serum amyloid A (Milan *et al*, 2012), and a large body of accumulated clinical evidence suggests that the VeriStrat Poor classification is associated with an aggressive disease state defined by host–tumour interactions. Clinical utility of the test was demonstrated, across various treatment regimens and indications, in a large number of retrospective studies (Carbone *et al*, 2010; Chung *et al*, 2010; Kuiper *et al*, 2012; Gautschi *et al*, 2013; Stinchcombe *et al*, 2013) and confirmed in a prospective phase III study, PROSE, that has shown the predictive role of the VeriStrat test in second-line NSCLC patients, that is, that patients classified as VeriStrat Poor gain significantly more benefit in terms of overall survival (OS) when treated with chemotherapy (docetaxel or pemetrexed) rather than erlotinib, whereas VeriStrat Good patients have similar OS in both regimens (Gregorc *et al*, 2014).

In addition, the prognostic properties of the test, that is, better outcomes associated with VeriStrat Good classification independent of treatment, were demonstrated in the retrospective analysis of samples from the placebo-controlled NCIC CTG BR.21 and TOPICAL studies (Carbone *et al*, 2012; Lee *et al*, 2015). Although being of significant clinical interest, at the time of study conception information on the role of the test in cytotoxic chemotherapy regimens was limited to several small data sets (Taguchi *et al*, 2007). To shed light on this problem, we designed a clinical trial to evaluate the role of VeriStrat as a biomarker in first-line standard chemotherapy for advanced NSCLC. Here we report on the performance of the test in patients with non-squamous histology treated with the combination of pemetrexed with cisplatin or carboplatin.

## Materials and methods

### Eligibility criteria

Chemotherapy-naive adults (18 years or older) with histologically or cytologically documented inoperable, locally advanced (stage IIIB with supraclavicular lymph node metastases), metastatic (stage IV) or recurrent NSCLC with life expectancy of more than 3 months, ECOG PS 0–2, at least one measurable lesion (as per response evaluation criteria in solid tumours (RECIST) criteria version 1.1), and adequate baseline bone marrow, hepatic, and renal functions were included in this study. Patients could have undergone prior radiation therapy (if completed before 28 days from study enrolment), or prior surgery (if completed before 14 days from study enrolment). Exclusion criteria were prior chemotherapy or treatment with other systemic anticancer agents, clinically significant cardiac disease, history or evidence of uncontrolled central nervous system disease, including brain metastases, active, or uncontrolled systemic disease or infection, as well as pregnancy or lactation.

All patients provided written informed consent; the trial was carried out in accordance with the Declaration of Helsinki and Good Clinical Practice guidelines and was approved by the Istituto Nazionale per la Ricerca sul Cancro ethics committee. The trial was registered at ClinicalTrials.gov (NCT02055144).

### Study treatment, design, and end points

This was an observational non-randomised study with prospective sample collection. The trial was designed to evaluate the role of VeriStrat in first-line chemotherapy in the real-world clinical setting, where patients were treated with platinum doublets according to the current guidelines and practices. Patients with squamous histology were treated with a combination of a platinum agent with gemcitabine; patients with non-squamous NSCLC received a combination of carboplatin (AUC 5) or cisplatin (75 mg/m^2^) with pemetrexed (500 mg/m^2^ q 21; Carbo/Pem and Cis/Pem regimens, respectively). The choice of platinum agent was at the physician's discretion based on age, ECOG PS, and creatinine clearance. Maintenance with pemetrexed (500 mg/m^2^ q 21 until progressive disease or discontinuation for toxicity) was allowed after four cycles of combination therapy, depending on response to the induction therapy, its toxicity, and ECOG PS. Imaging with computed tomography scans was performed at baseline and every 6 weeks (the equivalent of every two cycles, on schedule). Serum samples were collected before each drug administration until patient withdrawal and were frozen at −80 °C, until used for mass spectrum generation for VeriStrat testing. In this paper we discuss the results obtained from spectra collected at baseline before commencement of treatment.

Progression-free survival (PFS) in the VeriStrat-defined groups was chosen as the primary end point to avoid the possible confounding effects of subsequent treatments. It was calculated from the date of start of chemotherapy to the date of progression or death from any cause, whichever occurred first, or to the date of last radiological assessment in the absence of progression or death. Secondary end points included OS and correlation of VeriStrat classifications with best response, as well as with EGFR or Kirsten rat sarcoma viral oncogene (KRAS) mutations and ALK rearrangements; OS was calculated from the date of start of chemotherapy to the date of death from any cause or to the date of last medical contact, in absence of death. Patients who were event-free at the last clinical assessment were censored. The radiological response was assessed by RECIST version 1.1. All clinical evaluations were made blinded to the VeriStrat classification. The trial was designed and analysed in accordance with the REMARK initiative recommendations (McShane *et al*, 2005).

### Spectrum acquisition and VeriStrat classification

The commercially available VeriStrat test was conducted by Biodesix according to the standard protocol described elsewhere (Taguchi *et al*, 2007, Carbone *et al*, 2012) blinded to all clinical and treatment data. The test utilises matrix-assisted laser desorption/ionisation time-of-flight mass spectrometry to assign a VeriStrat Good or VeriStrat Poor classification to a serum or plasma sample by comparison of the intensity of eight regions in the spectra with the intensity of those of a reference set. Each patient sample is analysed in triplicate and VeriStrat labels are assigned if all three replicas result in the same classification; if they are discordant the sample is classified as indeterminate. There were no baseline indeterminate classifications assigned in this study.

### Other biomarker measurements

Assessments of EGFR and KRAS mutational statuses were performed with real-time PCR Therascreen IVD (Qiagen, Germantown, MD, USA) on formalin-fixed paraffin-embedded (FFPE) tissue. Chromosomal translocations involving the ALK gene were assessed using fluorescence *in situ* hybridisation using the Vysis dual colour Break Apart FISH Probe kit (Abbott Molecular, Abbott Park, IL, USA) on FFPE samples.

### Statistical plan and analyses

At the planning stage of the trial, we had no data on performance of VeriStrat in advanced NSCLC patients treated with first-line chemotherapy; however, we expected 30% of patients to be classified as VeriStrat Poor at baseline. The planned accrual time was 12 months, with an additional follow-up of 12 months, with 50 patients with non-squamous and 50 patients with squamous histology to be enroled. Assuming a power of 0.8, and a Type I error probability associated with testing of a null hypothesis, that the PFS of VeriStrat Poor and VeriStrat Good groups are equal, of 0.10, we estimated the detection limit of true hazard ratio (HR) for VeriStrat Good subjects relative to VeriStrat Poor subjects within each histology subtype of 0.46. The preliminary results of the trial were reported for the pre-planned number of non-squamous patients (*N*=55), demonstrating significant difference in PFS between VeriStrat Good and Poor patients overall and in the Carbo/Pem subgroup; however, in the Cis/Pem subgroup the difference did not reach statistical significance (Grossi *et al*, 2014). To confirm the preliminary results, it was decided to increase the power of the analysis by recruiting up to 90 patients with non-squamous histology over extended accrual period which, allowing for an attrition rate of 10–15% and indeterminate classification of 1–2%, and assuming equal distributions between treatment subgroups, would allow the detection of a HR of 0.43 in treatment subgroups and of 0.54 overall (with the same power and type 1 error, and accrual time increased to 36 months).

Time-to-event outcomes were analysed using data from patients who received at least one dose of chemotherapy and were classified as VeriStrat Good or VeriStrat Poor. Progression-free survival and OS were described by the Kaplan–Meier method and compared by log-rank test using GraphPad Prism 6 (La Jolla, CA, USA); the difference between groups was also assessed with unadjusted and adjusted Cox proportional hazard models for HRs, 95% confidence intervals (CI), and *P*-values using SAS Enterprise Guide 5.1 (Cary, NC, USA).

Patients' clinical characteristics are presented as the median and range for continuous variables and counts and percentages for discrete variables. *P*-values for association of categorical variables were calculated by Fisher's exact test, using SAS Enterprise Guide or Prism. Comparison of age between VeriStrat groups was performed using the unpaired *t*-test.

## Results

### Patient disposition and baseline characteristics

**Figure 1:**
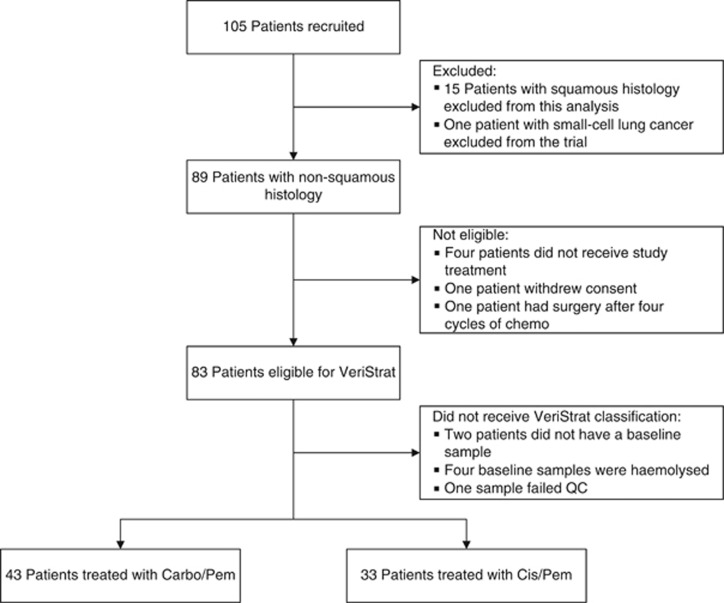
**CONSORT diagram.** Abbreviations: Cis/Pem=Cisplatin/Pemetrexed; Carbo/Pem=Carboplatin/Pemetrexed.

From May 2011 to October 2015, 90 patients with non-squamous histology and 15 patients with squamous histology entered the study. The accrual period was prolonged to support the increased, according to the updated statistical plan, number of patients eligible for platinum-based therapy. The number of patients with squamous histology was insufficient for analysis because of the low number of these patients eligible for platinum doublets within the population referred to the trial lung cancer unit, and they were not included in the current analysis. One patient with small cell lung cancer, one patient who withdrew consent, four patients not receiving the study treatment, and one patient who had an excellent response after four cycles of chemotherapy and was consequently treated with surgery were excluded. The remaining 83 patients were considered eligible for VeriStrat analysis. Out of those, two patients did not provide baseline samples, four patients had haemolysed baseline samples, and one sample failed the quality control. Seventy-six patients received baseline VeriStrat classifications: 43 patients were treated with Carbo/Pem, 33 – with Cis/Pem; 50 (66%) patients were classified as VeriStrat Good; and 26 (34%) were classified as VeriStrat Poor. The Consort diagram of the trial is shown in Figure 1.

Supplementary Table 1 shows the distribution of clinical characteristics between the treatment arms. Patients treated with Cis/Pem were significantly younger than patients treated with Carbo/Pem (median age: 57 years *vs* 70 years, *t*-test *P*<0.0001); apart from age, patients in both treatment arms had similar clinical characteristics.

Table 1 presents baseline characteristics of patients overall and by VeriStrat classification. The median age of patients was 66 years (range: 44–80 years); most patients were former or current smokers and had ECOG PS 1. Four patients had undergone prior radiation therapy and six had prior surgery. All patients had stage IV disease. Only one patient had a known ALK rearrangement, two patients had EGFR mutations (one in exon 21 and one in exon 20; the patient with exon 20 EGFR mutation was not treated with EGFR TKI; the patient with exon 21 EGFR mutation (L858R) received EGFR TKI in second line), and 28 had KRAS mutations (27 in exon 12 and 1 in exon 13); no correlations between mutation status and VeriStrat classification were observed, although the number of EGFR mutations and ALK rearrangements was very small.

There was no significant association between baseline clinical characteristics and VeriStrat classification; however, there were more patients receiving maintenance therapy in the VeriStrat Good group than in the VeriStrat Poor group (*P*=0.0065).

### Outcome Measures and the Role of VeriStrat

#### PFS and OS

By the time of the database lock, 68 patients (89%) experienced PFS events (43 in the VeriStrat Good and 25 in the VeriStrat Poor groups) and 55 patients (72%) had died (33 and 22 in VeriStrat Good and Poor groups, respectively). In the overall population, the median PFS was 3.8 months (95% CI 2.7–5.7), whereas the median OS was 7.9 month (95% CI 5.7–10.8); the median follow-up time was 26.2 months.

**Figure 2:**
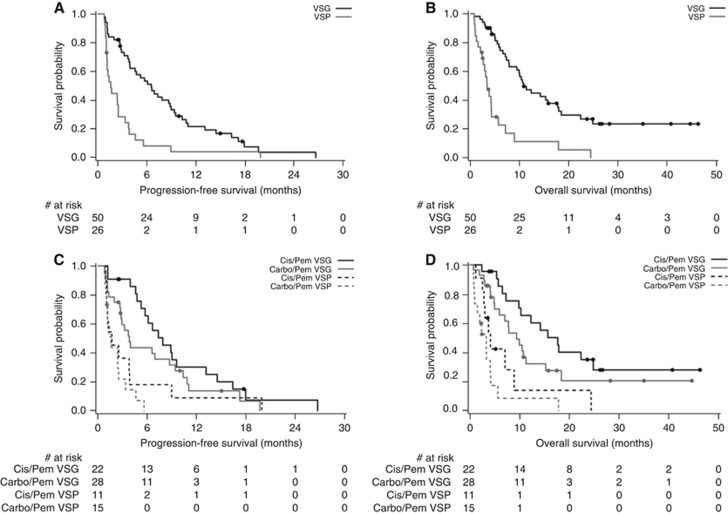
**Kaplan–Meier curves of PFS and OS.** (**A**) Progression-free survival by VeriStrat in overall population. (**B**) Overall survival by VeriStrat in overall population. (**C**) Progression-free survival by VeriStrat in treatment subgroups. (**D**) Overall survival by VeriStrat in treatment subgroups. Abbreviations: VSG=VeriStrat Good; VSP=VeriStrat Poor.

Both PFS and OS were significantly longer in patients classified as VeriStrat Good, as illustrated by the Kaplan–Meier curves (Figure 2A and B). The median PFS in the VeriStrat Good patients *vs* VeriStrat Poor patients was 6.5 *vs* 1.6 months (HR=0.36, 95% CI 0.22–0.61, *P*<0.0001); the median OS was 10.8 months *vs* 3.4 months (HR=0.26, 95% CI 0.15–0.47, *P*<0.0001), see Table 2. VeriStrat was prognostic in the multivariate analysis adjusted for clinical characteristics with *P*=0.0002 and <0.0001 for PFS and OS, respectively (Table 3A). When treatment regimen and maintenance status were added to the Cox proportional hazard model, VeriStrat remained significant (*P*=0.0019 for PFS and *P*<0.0001 for OS; Table 3B), indicating that the observed differences between VeriStrat Good and Poor patients are not just due to differences in maintenance or treatment regimen between these two groups.

These findings remained consistent in the exploratory subgroup analysis by treatment regimen: VeriStrat Good patients had a statistically significant advantage over VeriStrat Poor in PFS and OS both in Carbo/Pem and Cis/Pem subgroups (Figure 2C and D and Table 2). In the Carbo/Pem subgroup, the median PFS was 3.8 and 1.6 months in VeriStrat Good and VeriStrat Poor, respectively (HR=0.30, 95% CI 0.14–0.62, *P*=0.0007), whereas the median OS was 9.4 and 3.4 months, respectively (HR=0.26 95% CI 0.12–0.54, *P*=0.0002); in the Cis/Pem subgroup, the median PFS was 7.9 months in the VeriStrat Good group and 1.7 months in the VeriStrat Poor group (HR=0.39, 95% CI 0.18–0.85, *P*=0.0141), whereas the median OS was 17.7 and 4.2 months, respectively (HR=0.25, 95% CI 0.10–0.62, *P*=0.0013). Numerical differences between the median PFS and OS by treatment regimen within VeriStrat classification groups reflect the better overall outcomes observed in the Cis/Pem arm *vs* the Carbo/Pem arm (Table 2 and Supplementary Figure 1A and B), which may be explained by the younger age of patients in the Cis/Pem subgroup and other factors influencing the choice of chemotherapy in this non-randomised trial.

#### Response

Objective response rate was higher in the VeriStrat Good group (*P*=0.0032). In fact, 31% of VeriStrat Good patients achieved an objective response (1 complete and 14 partial), whereas there were no responses in the VeriStrat Poor group; 27 and 7 VeriStrat Good patients had stable disease and disease progression, respectively; in the VeriStrat Poor group there were 13 patients with stable disease and 9 patients with progressive disease. One VeriStrat Good patient and four VeriStrat Poor patients died before the first radiological assessment (see Table 4).

## Discussion

The advent of the third generation of cytotoxic agents led to some improvements in survival of patients affected by advanced NSCLC; however, the prognosis for these patients is still dire, the choice of an optimal treatment strategy remains challenging, and relevant biomarkers are needed, especially when targetable oncogenic drivers, such as EGFR or ALK, are missing. The most notable progress achieved in treatment of advanced NSCLC in the last years is associated with immunotherapy, in particular with development and approval by the Food and Drug Administration of the immune checkpoint inhibitors for previously treated advanced NSCLC patients. However, in the first line not all patients achieve greater clinical benefit from immunotherapies compared with platinum doublets, as the recent failure of the Checkmate 026 trial to show the advantage of nivolumab *vs* platinum-based chemotherapy in terms of PFS, has demonstrated despite the fact that patients were selected for positive PD-L1 expression (http://investor.bms.com/investors/news-and-events/press-releases/press-release-details/2016/Bristol-Myers-Squibb-Announces-Top-Line-Results-from-CheckMate—026-a-Phase-3-Study-of-Opdivo-nivolumab-in-Treatment-Nave-Patients-with-Advanced-Non-Small-Cell-Lung-Cancer/default.aspx, accessed 08/5/2016). Although it is possible that immunotherapy will be used in some NSCLC patients (West, 2014), it is most likely that traditional chemotherapy still remains a viable option for many patients with advanced disease. Currently, none of the studied biomarkers for cytotoxic therapy is employed in broad clinical practice for reasons ranging from insufficient clinical validation, to large variations in evaluation procedures and heterogeneity of the tumours, to the principal limitations of single-molecule measurements as biomarkers of complex biological processes (Malottki *et al*, 2016; Souglakos, 2015; Toffart *et al*, 2014).

VeriStrat, being a multivariate blood-based proteomic test that relates to the state of the whole organism, is better suited to overcome the limitations of single-molecule measurements to reflect complex biological processes of tumour–treatment-and–host interactions. The test is highly reproducible, provides rapid results from a non-invasive blood draw, and has been validated in multiple studies, including a prospective randomised phase III trial PROSE (Gregorc *et al*, 2014). It is commercially available and can be rapidly adopted in new indications.

Forty per cent of patients with newly diagnosed NSCLC have stage IV disease, and ∼70% of these patients are affected by non-squamous histology. The cohort analysed in this study is representative of this clinically important population of patients able to receive platinum-based chemotherapy: non-squamous NSCLC, ECOG PS 0–1, stage IV; 5% of patients had prior radiation, 8% had surgery, all of them treated with a combination of a platinum agent and pemetrexed with 42% continuing on maintenance therapy with pemetrexed after first line. The small number of enroled patients with ECOG PS 2 reflects the relatively low proportion of these patients in our practice deemed fit for a platinum-based regimen.

In this study, the choice between platinum agents was based on creatinine levels, age, and other clinical characteristics. Patients treated with Cis/Pem were younger, which reflects the common practice of prescribing cisplatin to fitter patients, and had longer PFS and OS than patients treated with Carbo/Pem, in agreement with previously published data (Ardizzoni *et al*, 2007; Moro-Sibilot *et al*, 2015), although the difference did not reach statistical significance. VeriStrat was able to identify patients who were more or less likely to have good outcomes from the platinum doublet in terms of PFS and OS in the overall population, as well as separately in the Carbo/Pem and Cis/Pem subgroups. Also notable is the absence of objective responses in patients classified as VeriStrat Poor, whereas 31% of VeriStrat Good patients had either complete or partial responses. In addition, as patients classified as VeriStrat Good were more likely to benefit from first-line chemotherapy in terms of survival and response, they were also more likely to receive maintenance with pemetrexed as single agent compared with those patients classified as VeriStrat Poor.

These results support our findings from the retrospective analysis of a similar cohort of patients with non-squamous NSCLC treated with a combination of cisplatin and gemcitabine in first line in the NExUS study, where VeriStrat Good classification was also associated with better prognosis, as well as with previously published data in the second line of treatment with erlotinib or chemotherapy from the PROSE trial (Gregorc *et al*, 2014) and erlotinib and placebo arms of the BR 21 study (Carbone *et al*, 2012) Interestingly, in the second arm of the NExUS study VeriStrat Poor patients treated with sorafenib in addition to platinum doublet had similar PFS to patients classified as VeriStrat Good (Vansteenkiste *et al*, 2012). Furthermore, when the role of VeriStrat was explored in patients treated with single agent gemcitabine, no significant difference between VeriStrat Good and Poor survival curves was found (Stinchcombe *et al*, 2013). These data, in combination with the results of the current study, suggest that the test may be predictive of differential outcomes between cytotoxic therapies. However, the limitation of this study is that in the absence of a control arm we could only show the prognostic effect of the test, that is, that patients classified as VeriStrat Good have better outcomes than those classified as VeriStrat Poor, and could not assess the predictive power with respect to differential benefit from some alternative treatment. A two-arm trial, which could extend the evidence of the predictive power of the test to the first-line setting and point to a better therapeutic option for VeriStrat Poor patients, is warranted. Nevertheless, facilitating patient understanding of the prognosis, independent of therapy, is critical for making informed decisions regarding treatment and end-of-life care (Enzinger *et al*, 2015).

In conclusion, this study met its objectives and demonstrated the clinical utility of VeriStrat as a prognostic marker in chemotherapy-naive patients with non-squamous NSCLC treated with standard chemotherapy regimens. In the era of genomic testing the utility of VeriStrat is in providing information complementary to that gained by an orthogonal methodology. The test can be useful for oncologists for planning the treatment strategy. Whereas patients with the VeriStrat Good classification have a better prognosis when treated with platinum doublets, classification as VeriStrat Poor may be an indication that alternative therapeutic options, including participation in clinical trials, should be explored. In addition, the results of the test may support an informed patient–physician discussion of the disease prognosis.

## Figures and Tables

**Table 1:** Baseline patient characteristics by VeriStrat classification

	**Overall**	**VeriStrat Good (*****N*****=50)**	**VeriStrat Poor (*****N*****=26)**	***P*****-value**
**Age (years)**				
Range	44–80	44–76	46–80	0.2639
Median	66	66	66	
**Gender**				
Male, *N* (%)	51 (67)	34 (68)	17 (65)	1
Female, *N* (%)	25 (33)	16 (32)	9 (35)	
**Histology**				
Adenocarcinoma, *N* (%)	75 (99)	49 (98)	26 (100)	1
NOS, *N* (%)	1 (1)	1 (2)	0	
**Stage**				
IV	76 (100)	50 (100)	26 (100)	—
**Smoking**				
Never smoker, *N* (%)	7 (9)	4 (8)	3 (11)	0.8219
Former smoker, *N* (%)	30 (40)	21 (42)	9 (35)	
Smoker, *N* (%)	39 (51)	25 (50)	14 (54)	
**Prior radiation therapy**				
No, *N* (%)	72 (95)	48 (96)	24 (92)	0.6028
Yes, *N* (%)	4 (5)	2 (4)	2 (8)	
**Prior surgery**				
No, *N* (%)	70 (92)	45 (90)	25 (96)	0.6576
Yes, *N* (%)	6 (8)	5 (10)	1 (4)	
**Maintenance**				
No, *N* (%)	44 (58)	23 (48)	21 (81)	0.0065
Yes, *N* (%)	32 (42)	27 (52)	5 (19)	
**ECOG PS**				
0, *N* (%)	20 (26)	15 (30.00)	5 (19)	0.4144
1, *N* (%)	54 (71)	33 (66.00)	21 (81)	
2, *N* (%)	2 (3)	2 (4.00)	0	
**Chemotherapy type**				
Carbo/Pem, *N* (%)	43 (57)	28 (56.00)	15 (58)	1
Cis/Pem, *N* (%)	33 (43)	22 (44.00)	11 (42)	
**KRAS status**				
Wild type, *N* (%)	31 (41)	24 (48.00)	7 (27)	0.1766
Mutation, *N* (%)	29 (38)	16 (32.00)	13 (50)	
Unknown, *N* (%)	16 (21)	10 (20.00)	6 (23)	
**EGFR status**				
Wild type, *N* (%)	67 (88)	43 (86.00)	24 (92)	0.855
Mutant, *N* (%)	2 (3)	2 (4.00)	0	
Unknown, *N* (%)	7 (9)	5 (10.00)	2 (8)	
**ALK translocation**				
Negative, *N* (%)	54 (71)	37 (74.00)	17 (65)	0.6193
Positive, *N* (%)	1 (1)	1 (2.00)	0	
Unknown, *N* (%)	21 (28)	12 (24.00)	9 (35)	

Abbreviations: ALK=anaplastic lymphoma kinase; Carbo=carboplatin; Cis=cisplatin; ECOG=Eastern Cooperative Oncology Group; EFGR=epidermal growth factor receptor; KRAS=Kirsten rat sarcoma viral oncogene; NOS=not otherwise specified; Pem=pemetrexed; PS=performance status.

**Table 2:** Time-to-event outcomes in VeriStrat- and treatment-defined groups of patients

	**PFS**			**OS**		
**Patient groups**	**Median (95% CI), month**	**HR (95% CI)**	**Log-rank** ***P***	**Median (95% CI), month**	**HR (95% CI)**	**Log-rank** ***P***
VSG	6.5 (3.9–8.8)	0.36^a^ (0.22–0.61)	<0.0001	10.8 (7.8–17.7)	0.26^a^ (0.15–0.47)	<0.0001
VSP	1.6 (1.1–2.5)			3.4 (2.4–4.3)		
VSG Carbo/Pem	3.8 (2.7–8.7)	0.30^a^ (0.14–0.62)	0.0007	9.4 (5.0–15.3)	0.26^a^ (0.12–0.55)	0.0002
VSP Carbo/Pem	1.6 (1.0–2.5)			3.4 (1.0–4.3)		
VSG Cis/Pem	7.9 (5.2–13.1)	0.39^a^ (0.18–0.85)	0.0141	17.7 (9.9–24.9)	0.25^a^ (0.10–0.62)	0.0013
VSP Cis/Pem	1.7 (1.1–3.9)			4.2 (2.6–8.9)		
Carbo/Pem	2.8 (2.0–4.0)	1.59^b^ (0.97–2.61)	0.0627	6.0 (4.2–10.0)	1.64^b^ (0.96–2.82)	0.0701
Cis/Pem	5.7 (3.8–8.8)			10.3 (6.6–17.9)		

Abbreviations: CI=confidence interval; Carbo=carboplatin; Cis=cisplatin; HR=hazard ratio; N/R=not reached; OS=overall survival; Pem=pemetrexed; PFS=progression-free survival; VSG=VeriStrat Good; VSP=VeriStrat Poor.

a HR calculated for VeriStrat Good *vs* VeriStrat Poor using unadjusted Cox proportional hazard model.

b HR calculated for Carbo/Pem *vs* Cis/Pem using unadjusted Cox proportional hazard model.

**Table 3:** Adjusted Cox proportional hazard analysis of PFS and OS

	**PFS**		**OS**	
	**HR (95% CI)**	***P*****-value**	**HR (95% CI)**	***P*****-value**
**Model including only clinical characteristics**				
VeriStrat classification (good *vs* poor)	0.32 (0.18–0.58)	0.0002	0.23 (0.12–0.44)	<0.0001
Gender (male *vs* female)	1.27 (0.72–2.24)	0.4028	1.58 (0.84–2.98)	0.1604
Smoking status (ever *vs* never)	1.09 (0.46–2.60)	0.8519	1.49 (0.58–3.81)	0.4062
ECOG PS (⩾1 *vs* 0)	1.10 (0.62–2.01)	0.7213	1.07 (0.54–2.12)	0.8414
KRAS status (mutant *vs* WT or unknown)	0.98 (0.54–1.80)	0.9505	1.21 (0.62–2.34)	0.5794
KRAS known (known *vs* unknown)	3.13 (1.41–6.95)	0.0049	2.87 (1.17–7.07)	0.0219
**Model including clinical characteristics and treatment**				
VeriStrat classification (good *vs* poor)	0.39 (0.22–0.71)	0.0019	0.23 (0.11–0.46)	<0.0001
Gender (male *vs* female)	1.36 (0.77–2.39)	0.2933	1.68 (0.87–3.23)	0.1220
Tx regimen (Cis/Pem *vs* Carbo/Pem)	1.87 (1.10–3.16)	0.0202	1.86 (1.05–3.32)	0.0343
Smoking status (ever *vs* never)	1.43 (0.58–3.56)	0.4396	2.73 (0.91–8.20)	0.0727
ECOG PS (⩾1 *vs* 0)	1.12 (0.61–2.03)	0.7204	1.00 (0.51–1.99)	0.9976
KRAS status (mutant *vs* WT or unknown)	1.11 (0.61–2.04)	0.7312	1.26 (0.64–2.47)	0.5032
KRAS (known *vs* unknown)	2.31 (1.06–5.07)	0.0363	1.90 (0.78–4.61)	0.1553
Maintenance (yes *vs* no)	0.35 (0.20–0.60)	0.0002	0.27 (0.14–0.52)	<0.0001

Abbreviations: CI=confidence interval; Carbo=carboplatin; Cis=cisplatin; ECOG=Eastern Cooperative Oncology Group; HR=hazard ratio; KRAS=Kirsten rat sarcoma viral oncogene; OS=overall survival; Pem=pemetrexed; PFS=progression-free survival; PS=performance status; Tx=treatment; WT=wild type.

**Table 4:** Objective response by VeriStrat status

	**VeriStrat Good**	**VeriStrat Poor**	***P*****-value**
PR/CR	1/14 (30.6%)	0 (0)	0.0032
PD/SD	7/27 (69.4%)	9/13 (100%)	
ED	1	4	

Abbreviations: CR=complete response; ED=early death; PD=progressive disease; PR=partial response; SD=stable disease.

## References

[bib1] Ardizzoni A, Boni L, Tiseo M, Fossella FV, Schiller JH, Paesmans M, Radosavljevic D, Paccagnella A, Zatloukal P, Mazzanti P, Bisset D, Rosell R (2007) Cisplatin- versus carboplatin-based chemotherapy in first-line treatment of advanced non-small-cell lung cancer: an individual patient data meta-analysis. J Natl Cancer Inst 99(11): 847–857. 17551145 10.1093/jnci/djk196

[bib2] Azuma K, Komohara Y, Sasada T, Terazaki Y, Ikeda J, Hoshino T, Itoh K, Yamada A, Aizawa H (2007) Excision repair cross-complementation group 1 predicts progression-free and overall survival in non-small cell lung cancer patients treated with platinum-based chemotherapy. Cancer Sci 98(9): 1336–1343. 17640298 10.1111/j.1349-7006.2007.00557.x PMC11158988

[bib3] Carbone DP, Ding K, Roder H, Grigorieva J, Roder J, Tsao MS, Seymour L, Shepherd FA (2012) Prognostic and predictive role of the VeriStrat plasma test in patients with advanced non-small-cell lung cancer treated with erlotinib or placebo in the NCIC Clinical Trials Group BR.21 trial. J Thorac Oncol 7(11): 1653–1660. 23059783 10.1097/JTO.0b013e31826c1155 PMC3728561

[bib4] Carbone DP, Salmon JS, Billheimer D, Chen H, Sandler A, Roder H, Roder J, Tsypin M, Herbst RS, Tsao AS, Tran HT, Dang TP (2010) VeriStrat classifier for survival and time to progression in non-small cell lung cancer (NSCLC) patients treated with erlotinib and bevacizumab. Lung Cancer 69(3): 337–340. 20036440 10.1016/j.lungcan.2009.11.019 PMC2891357

[bib5] Chung CH, Seeley EH, Roder H, Grigorieva J, Tsypin M, Roder J, Burtness BA, Argiris A, Forastiere AA, Gilbert J, Murphy B, Caprioli RM, Carbone DP, Cohen EE (2010) Detection of tumor epidermal growth factor receptor pathway dependence by serum mass spectrometry in cancer patients. Cancer Epidemiol Biomarkers Prev 19(2): 358–365. 20086114 10.1158/1055-9965.EPI-09-0937 PMC2846615

[bib6] Delbaldo C, Michiels S, Rolland E, Syz N, Soria JC, Le Chevalier T, Pignon JP (2007) Second or third additional chemotherapy drug for non-small cell lung cancer in patients with advanced disease. Cochrane Database Syst Rev, (4): CD004569. 17943820 10.1002/14651858.CD004569.pub2

[bib7] Enzinger AC, Zhang B, Schrag D, Prigerson HG (2015) Outcomes of prognostic disclosure: associations with prognostic understanding, distress, and relationship with physician among patients with advanced cancer. J Clin Oncol 33(32): 3809–3816. 26438121 10.1200/JCO.2015.61.9239 PMC4737862

[bib8] Gautschi O, Dingemans AM, Crowe S, Peters S, Roder H, Grigorieva J, Roder J, Zappa F, Pless M, Brutsche M, Baty F, Bubendorf L, Hsu Schmitz SF, Na KJ, Carbone D, Stahel R, Smit E (2013) VeriStrat(R) has a prognostic value for patients with advanced non-small cell lung cancer treated with erlotinib and bevacizumab in the first line: pooled analysis of SAKK19/05 and NTR528. Lung Cancer 79(1): 59–64. 23122759 10.1016/j.lungcan.2012.10.006

[bib9] Gregorc V, Novello S, Lazzari C, Barni S, Aieta M, Mencoboni M, Grossi F, Pas TD, de Marinis F, Bearz A, Floriani I, Torri V, Bulotta A, Cattaneo A, Grigorieva J, Tsypin M, Roder J, Doglioni C, Levra MG, Petrelli F, Foti S, Vigano M, Bachi A, Roder H (2014) Predictive value of a proteomic signature in patients with non-small-cell lung cancer treated with second-line erlotinib or chemotherapy (PROSE): a biomarker-stratified, randomised phase 3 trial. Lancet Oncol 15(7): 713–721. 24831979 10.1016/S1470-2045(14)70162-7

[bib10] Grossi F, Genova C, Rijavec E, Bello MGD, Barletta G, Burrafato G, Biello F, Sini C, Grigorieva J, Meyer K, Roder H (2014) Serum mass-spectrometry test in first line advanced NSCLC patients treated with standard chemotherapy regimens. Ann Oncol 25(suppl 4): iv426–iv470.

[bib11] Kuiper JL, Lind JS, Groen HJ, Roder J, Grigorieva J, Roder H, Dingemans AM, Smit EF (2012) VeriStrat((R)) has prognostic value in advanced stage NSCLC patients treated with erlotinib and sorafenib. Br J Cancer 107(11): 1820–1825. 23079575 10.1038/bjc.2012.470 PMC3505013

[bib12] Lee SM, Nash S, Ngai Y, Hackshaw A (2015) Prognostic and predictive value of the veristrat classifier in chemo-naive NSCLC patients treated with erlotinib or placebo (TOPICAL Trial). In 16th World Conference on Lung Cancer (WCLC): 6–9 September 2015, Denver, CO, ID 699.

[bib13] Malottki K, Popat S, Deeks JJ, Riley RD, Nicholson AG, Billingham L (2016) Problems of variable biomarker evaluation in stratified medicine research-A case study of ERCC1 in non-small-cell lung cancer. Lung Cancer 92: 1–7. 26775588 10.1016/j.lungcan.2015.11.017 PMC4729317

[bib14] Martin LP, Hamilton TC, Schilder RJ (2008) Platinum resistance: the role of DNA repair pathways. Clin Cancer Res 14(5): 1291–1295. 18316546 10.1158/1078-0432.CCR-07-2238

[bib15] Masters GA, Temin S, Azzoli CG, Giaccone G, Baker S Jr, Brahmer JR, Ellis PM, Gajra A, Rackear N, Schiller JH, Smith TJ, Strawn JR, Trent D, Johnson DH (2015) Systemic therapy for stage IV Non-small-cell lung cancer: American Society of Clinical Oncology Clinical Practice Guideline Update. J Clin Oncol 33(30): 3488–3515. 26324367 10.1200/JCO.2015.62.1342 PMC5019421

[bib16] McShane LM, Altman DG, Sauerbrei W, Taube SE, Gion M, Clark GM (2005) REporting recommendations for tumour MARKer prognostic studies (REMARK). Eur J Cancer 41(12): 1690–1696. 16043346 10.1016/j.ejca.2005.03.032

[bib17] Milan E, Lazzari C, Anand S, Floriani I, Torri V, Sorlini C, Gregorc V, Bachi A (2012) SAA1 is over-expressed in plasma of non small cell lung cancer patients with poor outcome after treatment with epidermal growth factor receptor tyrosine-kinase inhibitors. J Proteomics 76: 91–101. 22771314 10.1016/j.jprot.2012.06.022

[bib18] Moro-Sibilot D, Smit E, de Castro Carpeno J, Lesniewski-Kmak K, Aerts J, Villatoro R, Kraaij K, Nacerddine K, Dyachkova Y, Smith KT, Taipale K, Girvan AC, Visseren-Grul C, Schnabel PA (2015) Outcomes and resource use of non-small cell lung cancer (NSCLC) patients treated with first-line platinum-based chemotherapy across Europe: FRAME prospective observational study. Lung Cancer 88(2): 215–222. 25748103 10.1016/j.lungcan.2015.02.011

[bib19] Nicolson MC, Fennell DA, Ferry D, O'Byrne K, Shah R, Potter V, Skailes G, Upadhyay S, Taylor P, Andre V, Nguyen TS, Myrand SP, Visseren-Grul C, Das M, Kerr KM (2013) Thymidylate synthase expression and outcome of patients receiving pemetrexed for advanced nonsquamous non-small-cell lung cancer in a prospective blinded assessment phase II clinical trial. J Thorac Oncol 8(7): 930–939. 23722170 10.1097/JTO.0b013e318292c500

[bib20] Reck M, Popat S, Reinmuth N, De Ruysscher D, Kerr KM, Peters S (2014) Metastatic non-small-cell lung cancer (NSCLC): ESMO Clinical Practice Guidelines for diagnosis, treatment and follow-up. Ann Oncol 25(Suppl 3): iii27–iii39. 25115305 10.1093/annonc/mdu199

[bib21] Ries LAG, Eisner MP, Kosary CL, Hankey BF, Miller BA, Clegg L, Mariotto A, Feuer EJ, Edwards BK (2005) In SEER Cancer Statistics Review pp 1975–2002. National Cancer Institute: Bethesda, MD.

[bib22] Rodrigues-Pereira J, Kim JH, Magallanes M, Lee DH, Wang J, Ganju V, Martinez-Barrera L, Barraclough H, van Kooten M, Orlando M (2011) A randomized phase 3 trial comparing pemetrexed/carboplatin and docetaxel/carboplatin as first-line treatment for advanced, nonsquamous non-small cell lung cancer. J Thorac Oncol 6(11): 1907–1914. 22005471 10.1097/JTO.0b013e318226b5fa

[bib23] Scagliotti GV, Parikh P, von Pawel J, Biesma B, Vansteenkiste J, Manegold C, Serwatowski P, Gatzemeier U, Digumarti R, Zukin M, Lee JS, Mellemgaard A, Park K, Patil S, Rolski J, Goksel T, de Marinis F, Simms L, Sugarman KP, Gandara D (2008) Phase III study comparing cisplatin plus gemcitabine with cisplatin plus pemetrexed in chemotherapy-naive patients with advanced-stage non-small-cell lung cancer. J Clin Oncol 26(21): 3543–3551. 18506025 10.1200/JCO.2007.15.0375

[bib24] Souglakos J (2015) Customizing chemotherapy in non-small cell lung cancer: the promise is still unmet. Transl Lung Cancer Res 4(5): 653–655. 26629440 10.3978/j.issn.2218-6751.2015.03.10 PMC4630530

[bib25] Stinchcombe TE, Roder J, Peterman AH, Grigorieva J, Lee CB, Moore DT, Socinski MA (2013) A retrospective analysis of VeriStrat status on outcome of a randomized phase II trial of first-line therapy with gemcitabine, erlotinib, or the combination in elderly patients (age 70 years or older) with stage IIIB/IV non-small-cell lung cancer. J Thorac Oncol 8(4): 443–451. 23370367 10.1097/JTO.0b013e3182835577

[bib26] Sun JM, Ahn JS, Jung SH, Sun J, Ha SY, Han J, Park K, Ahn MJ (2015) Pemetrexed plus cisplatin versus gemcitabine plus cisplatin according to thymidylate synthase expression in nonsquamous non-small-cell lung cancer: a biomarker-stratified randomized phase II trial. J Clin Oncol 33(22): 2450–2456. 26124486 10.1200/JCO.2014.59.9324

[bib27] Taguchi F, Solomon B, Gregorc V, Roder H, Gray R, Kasahara K, Nishio M, Brahmer J, Spreafico A, Ludovini V, Massion PP, Dziadziuszko R, Schiller J, Grigorieva J, Tsypin M, Hunsucker SW, Caprioli R, Duncan MW, Hirsch FR, Bunn PA Jr., Carbone DP (2007) Mass spectrometry to classify non-small-cell lung cancer patients for clinical outcome after treatment with epidermal growth factor receptor tyrosine kinase inhibitors: a multicohort cross-institutional study. J Natl Cancer Inst 99(11): 838–846. 17551144 10.1093/jnci/djk195

[bib28] Toffart AC, Timsit JF, Couraud S, Merle P, Moro-Sibilot D, Perol M, Mastroianni B, Souquet PJ, Girard N, Jeannin G, Romand P, Chatellain P, Vesin A, Brambilla C, Brambilla E (2014) Immunohistochemistry evaluation of biomarker expression in non-small cell lung cancer (Pharmacogenoscan study). Lung Cancer 83(2): 182–188. 24388706 10.1016/j.lungcan.2013.12.003

[bib29] Vansteenkiste J, Paz-Ares L, Eisen T, Heigener D, Eberhardt R, Thomas M, Zhou C, Santoro A, Lathia C, Roder H (2012) A plasma proteomic signature predicts outcomes in a Phase 3 study of gemcitabine (G)+cisplatin (C)±sorafenib in first line Stage IIIB or IV NSCLC. Ann Oncol 23(suppl 9): ix407.

[bib30] West H (2014) Nivolumab as first line monotherapy for advanced non-small cell lung cancer: could we replace first line chemotherapy with immunotherapy? Transl Lung Cancer Res 3(6): 400–402. 25806333 10.3978/j.issn.2218-6751.2014.09.04 PMC4367667

