# Supplementary figures and images for: Serum proteomic test in advanced non-squamous non-small cell lung cancer treated in first line with standard chemotherapy
Source: Br J Cancer. 2016 Nov 29;116(1):36–43. doi: 10.1038/bjc.2016.387 (PMC5220151; doi:10.1038/bjc.2016.387)

## Slide 1
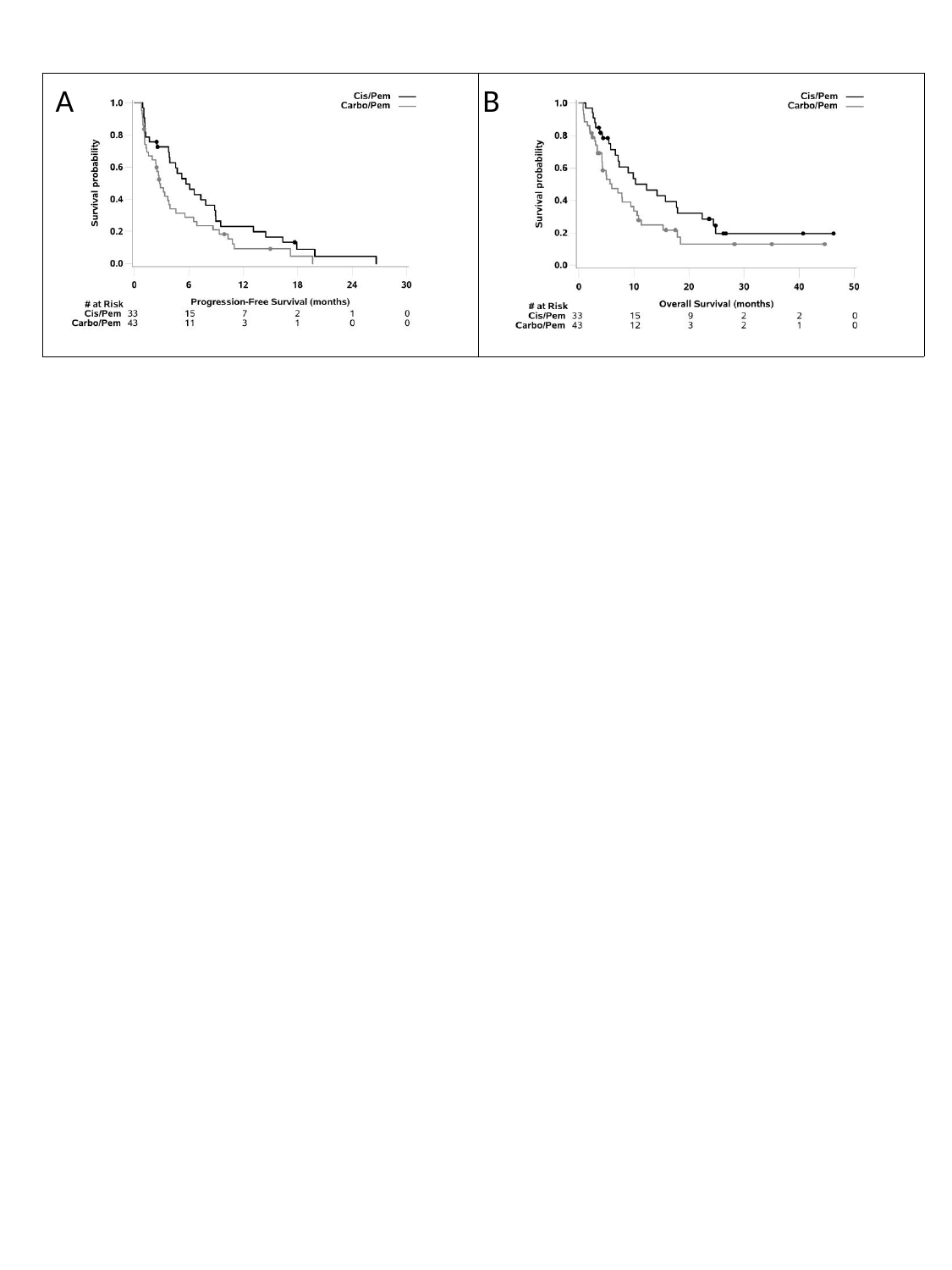

| | |
| --- | --- |
A B

Supplement: Supplementary Figure 1 [file bjc2016387x2.ppt]
