# Supplementary material for: Serum proteomic test in advanced non-squamous non-small cell lung cancer treated in first line with standard chemotherapy
Source: Br J Cancer. 2016 Nov 29;116(1):36–43. doi: 10.1038/bjc.2016.387 (PMC5220151; doi:10.1038/bjc.2016.387)
Supplement: Supplementary Information [file bjc2016387x3.docx]

# Supplementary material

**Content:**

SUPPLEMENTAL TABLE 1. Baseline Patient Characteristics by Chemotherapy Regimen

File: Grossi et al_Supplement TABLE1.xlsx

SUPPLEMENTAL FIGURE 1. **Kaplan-Meier curves of PFS (A) and OS (B) by chemotherapy**

Abbreviations: Cis/Pem, Cisplatin/Pemetrexed; Carbo/Pem, Carboplatin/Pemetrexed

File: Grossi_SUPPLEMENT_Fig1_KM curves of PFS and OS_chemo regimens.pptx
